# Gaming among female adolescents: profiling and psychopathological characteristics in the Indian context

**DOI:** 10.3389/fpsyt.2023.1081764

**Published:** 2023-05-05

**Authors:** Pranjali Chakraborty Thakur, Manoj Kumar Sharma, Vineeth Mohan, John Vijay Sagar Kommu, Nitin Anand, Palaniappan Marimuthu

**Affiliations:** ^1^Service for Healthy Use of Technology (SHUT) Clinic, Department of Clinical Psychology, National Institute of Mental Health and Neuro-sciences (NIMHANS), Bengaluru, Karnataka, India; ^2^Department of Clinical Psychology, National Institute of Mental Health and Neuro-sciences (NIMHANS), Bengaluru, Karnataka, India; ^3^Department of Clinical Neurosciences, National Institute of Mental Health and Neuro-sciences (NIMHANS), Bengaluru, Karnataka, India; ^4^Department of Child and Adolescent Psychiatry, National Institute of Mental Health and Neuro-sciences (NIMHANS), Bengaluru, Karnataka, India; ^5^Department of Biostatistics, National Institute of Mental Health and Neuro-sciences (NIMHANS), Bengaluru, Karnataka, India

**Keywords:** gaming, psychopathology, Internet Gaming Disorder, female adolescents, self-esteem, conduct, peer problems

## Abstract

**Objectives:**

Gaming is a predominant leisure time activity among adolescents, and the literature suggests that unrestrained gaming behavior might lead to gaming disorder. ICD-11 and DSM-5 have recognized gaming disorder as a psychiatric condition and grouped it under the behavioral addiction category. Research on gaming behavior and addiction is largely based on data from the male population, and problematic gaming has largely been understood from the male perspective. In this study, we are attempting to bridge the existing lacuna in the literature by exploring gaming behavior, gaming disorder, and its related psychopathological characteristics among female adolescents in India.

**Methods:**

The study was conducted on a sample of 707 female adolescent participants who were contacted through schools and academic institutes in a city in Southern India. The study adopted a cross-sectional survey design, and data were administered using the mixed modality of online and offline data collection. The participants filled out the following set of questionnaires: socio-demographic sheet, Internet Gaming Disorder Scale-Short-Form (IGDS9-SF), Strength and Difficulties Questionnaire (SDQ), Rosenberg self-esteem scale, and Brief sensation-seeking scale (BSSS-8). The data gathered from the participants were then statistically analyzed using SPSS software version 26.

**Results:**

The descriptive statistics revealed that 0.8% of the sample (i.e., five participants out of 707) obtained scores meeting gaming addiction criteria. Correlation analysis demonstrated a significant relationship between all the psychological variables with total IGD scale scores (*p* < 0.05). Total SDQ, total BSSS-8, and domain scores of SDQ, such as emotional symptoms, conduct, hyperactivity, and peer problems, were positively correlated, whereas total Rosenberg scores and domain scores of prosocial behaviors of SDQ were negatively correlated. The Mann–Whitney *U*-test was employed to compare “with gaming disorder” and “without gaming disorder” categories of female participants. Comparing these two groups revealed significant differences in emotional symptoms, conduct, hyperactivity/inattention, peer problem, and self-esteem scale scores. Furthermore, quantile regression was computed, showing that conduct, peer problem, and self-esteem displayed trend-level prediction for gaming disorder.

**Conclusion:**

Female adolescents prone to gaming addiction can be identified through psychopathological characteristics of conduct, peer problem, and low self-esteem. This understanding can be useful in developing a theoretical model focusing on early screening and preventive strategies for at-risk female adolescents.

## 1. Introduction

Gaming activities have become widely popular with the advent of digital media and technology. However, increased gaming behavior remains a risk for pathological use (1, 2). Gaming patterns, behavior, and their effect on mental health have gained considerable attention from psychiatric and psychosocial perspectives for decades (3). The history of research focusing on internet use, its application such as gaming, and associated functional problems can be dated back to the 1990's (4). However, recently, issues related to gaming behavior have been accorded a clinical status.

The American Psychiatric Association (APA) officially included Internet Gaming Disorder in its fifth edition of the Diagnostic and Statistical Manual of Mental Disorders (DSM-5) as a condition for further study and placed it under the section of “non-substance use” alongside gambling disorder, whereas the World Health Organization (WHO) enlisted gaming disorder (GD) as a psychiatric condition in the International Classification of Diseases (ICD-11) under the category of behavioral addiction (5–7).

DSM-5 identified nine clinical symptoms of IGD- preoccupation, loss of control, withdrawal and tolerance, loss of interest in other activities, continued use despite conflict, feeling of escape from reality, and impairment of social functioning (5, 7). ICD-11, on the other hand, is characterized by gaming disorder by only three symptoms: impaired control over gaming, increasing priority given to gaming, and continuation or escalation of gaming despite impairment (8).

Research on etiology has generally viewed IGD from a multidimensional perspective, encompassing neurobiological and psychosocial factors (9). Neurobiological investigations tend to support the idea that IGD may have an “addiction” component. Research using the cue-induced paradigm in IGD found that the same brain areas that are more active in substance abusers were also active more frequently among gaming addicts. Participants with IGD were found to have altered response inhibition functions, which is a pattern resembling that of substance use disorders (10, 11).

Psychological variables linked to IGD are the need to escape reality through gaming, way of coping with relieving stress, mood modulation, novelty seeking, and lack of social skills. A few social determinants such as social isolation, family conflict, a lack of acceptance of gaming as a problem, and fear of stigmatization also contributed to the condition (12).

A recent meta-analysis study examining the prevalence of Internet Gaming Disorder (IGD) among the general population from 2009 to 2019 was done. The studies were selected across 17 countries, mostly European, and many of them included only adolescents. The main findings showed the global pooled prevalence of IGD was 3.05%, present mostly among adolescents and young adults compared to older people. The prevalence ratio was much higher in male subjects than female subjects (13). Another global prevalence study in 2020 estimated that the percentage range of IGD varied from 0.21 to 58% in general populations and from 3.20 to 91% among clinical populations. The prevalence rates were higher among Asian countries compared to non-Asian countries; however, the prevalence record for the South-East Asian region was not available (14). The prevalence of IGD in the Indian population among school, undergraduate, and postgraduate students ranged from 1.3 to 19.9% for the adolescent group and 4% for young adults (15, 16). The ratio of male gamers with the disorder is higher than female gamers. During the pandemic, physical restrictions and lack of stimulation increased online activity, particularly gaming-related activity, which may have posed a higher risk for IGD (17, 18).

Based on the review studies, it can be concluded that the prevalence of gaming disorder is higher among the adolescent age group, especially in the male population (13, 14, 16, 19, 20). However, in recent times, the trend of the increasing popularity of gaming among female gamers has been observed (21). The International Software Federation of Europe reported 47% of European gamers were women. Similarly, data reported among the American gaming population stated that 45% were female gamers (22). Female gamers play games to look for competition, achievement, and appreciation through succeeding (23), gain power and self-esteem, engage socially, meet like-minded people on gaming forums, and maintain relationships with other gamers (24, 25). They perceive numerous physical and cognitive benefits of gaming, including mobility, dexterity, executive functions, and a sense of wellbeing (26). In summary, the desire for gaming among female gamers relates to several factors, including achievement, social needs, perceived hostility, and self-worth in the gaming community (23).

Studies show women start playing games just as frequently as male gamers do, and they have the same abilities to succeed at a similar level as them but because of gender expectations and peer community perception, women stop playing earlier in the gaming trajectory (27). Most violent video games display a sexualized representation of women, discouraging women from playing these games as it leads to negative body image and lowers their confidence (21, 25). Additionally, women also face regular harassment while playing as they are considered less skilful and competent by their male counterparts, and the fact that they are a minority in the gaming community (24). Subsequently, the ruminations of harassment, feeling of helplessness due to unresponsiveness to the issue, and persistent negative online experiences reduce their enjoyment and cause women to withdraw from the gaming world (25).

Regarding gaming types, female gamers prefer casual games, typically of shorter periods, or games that allow them to engage in narratives and virtual esthetics (21, 24). The most common gaming genre, on the other hand, is the violent first-person shooter genre, which male players primarily choose as it allows them to express their aggressive tendencies. Unfortunately, the gaming industries design video games that cater to the preferences of the male population (26), and a similar trend is also reflected in research on gaming. The gaming industry and our understanding of the gaming world have been largely male-focused, and this might explain why women are underrepresented in the gaming industry.

Female gamers are gradually taking up space in the online gaming community. Their status in competitive games has also changed from non-skilled players to moderately skilled players over the years (28). In the literature, the knowledge of female gaming is fairly underdeveloped (25). There is limited data on female gaming patterns, profiles, and risk factors, but some research studies show that female gamers might be at risk of problematic gaming (24). Most studies focused on male psychopathology associated with competitive gaming (12, 29), and data on problematic female gamers is almost non-existent (5).

Many studies have identified a few risk factors for addicted male gamers, such as impulsivity, addiction, anxiety, and depression (30, 31). Nonetheless, psychological problems have also been noted among addicted female gamers (32). In this study, we intend to explore gaming patterns among female adolescents and whether emotional and behavioral problems among female subjects are associated with problematic gaming.

The study aimed to assess the association of problematic gaming with emotional and behavioral difficulties among female adolescents. The study's objectives are (i) to identify the trends and patterns of Internet gaming among females and (ii) to assess the role of emotional and behavioral difficulties in female gamers as a predictive factor for problematic gaming.

## 2. Materials and methods

### 2.1. Participants and procedure

The study was conducted on 707 female adolescents from March 2021 to February 2022 in the Southern region of India. The sample belonged to the age range of 12–19 years, based on the WHO definition of adolescents. The participants in the study were recruited from day boarding schools (government and private institutes), and they all could read, write, and speak in English. All the scales were administered in the English language. Any participant with a health condition that could interfere with engaging in the study or unwilling to provide consent was excluded from the research.

The data were collected using a convenience-based sampling strategy. The researcher requested permission to conduct the study by contacting the management of multiple schools in the city of Bangalore in Southern India. The schools that agreed to participate in the research were then informed about the nature of the study, and specific classes from grades 8 to 12 were selected based on convenience among those schools.

Tools were administered using a mixed mode of online as well offline methods. During that period, many schools were closed for physical classes due to the pandemic and were operating via online mediums. The online mode was conducted by uploading the digital survey on the school's portal. The questionnaires were physically distributed among participants during a designated class time for the offline mode.

Ethical permission to conduct the research was obtained from the researcher's institute National Institute of Mental Health and Neuro-Sciences (NIMHANS). After briefing them about the research, the researcher obtained informed assent and informed consent from the participants and parents. No compensation or remuneration was offered for participation in the study, and this information was communicated to the participants in advance. A total number of 707 female adolescents participated in the research. However, complete data on scales of gaming addiction and other psychological variables were obtained from 589 female participants. The data obtained were subjected to statistical analysis.

### 2.2. Measures

#### 2.2.1. Socio-demographic sheet

This tool enquired basic information about the participants' age, grade, socio-economic status, family type, whether they are the single child in the family, occupational status of parents, school performance, relationship with teachers and classmates, engagement in extra-curricular activities, the experience of substance use and whether they have any prevailing physical or mental health condition (see Table 1). Specific information about gaming behavior and its impact on their lifestyle and daily functioning was also investigated. Interpersonal factors (friendships and perceived environment in school) and intrapersonal factors (such as loneliness, boredom, and unstructured free time) were inquired about.

**Table 1:** Socio-demographic details.

**Variable (*N* = 707)**	
**Age (years)**	
Mean ± SD	15.01 ± 1.55
Median (min, max)	16 (12, 19)
Both parents employed	293 (41.4%)
Single child	131 (18.5%)
**Type of family**	
Nuclear family	488 (69.0%)
Joint family	156 (22.1%)
Single parent	57 (8.1%)
**Monthly income**	
< USD 600	330 (46.7%)
USD 600–2,428	277 (39.2%)
>USD 2,428	69 (9.8%)
**Substance use**	
Never	665 (94.1%)
Sometimes	15 (2.1%)
Mostly	17 (2.4%)
History of physical health problems	25 (3.5%)
History of mental health problems	21 (3.0%)
**Gaming frequency per week**	
Never	286 (40.5%)
1–3 times a week	240 (33.9%)
4–5 times a week	39 (5.5%)
Everyday	45 (6.4%)
**Gaming duration (hours) per day**	
< 2 h	366 (51.8%)
2–6 h	15 (2.1%)
>6 h	2 (0.3%)

#### 2.2.2. Strength and difficulties questionnaire

This brief emotional and behavioral screening questionnaire for children and young people was used, as developed by Robert N. Goodman in 2001 (33). The scale has five subscales, namely, “emotional symptoms,” “conduct problems,” “hyperactivity and attention deficit problems,” “peer relationship problems,” and “prosocial behavior.” Each subscale comprises five items with a total of 25 items and uses 3-point Likert questions. Scores ranged from 0 (not true) to 2 (certainly true). The higher score in each sub-scale indicates a higher level of the psychological variable. In this study, Cronbach's alpha for the scale was 0.694, indicating adequate reliability.

#### 2.2.3. Internet Gaming Disorder Scale-Short-Form (IGDS9-SF)

This scale was formed by Pontes and Griffiths in 2015 (34), and it assesses the severity of Internet Gaming Disorder and its detrimental effects occurring over 12 months. The scale comprises nine items corresponding to the nine-core criteria defined by the DSM-5. They are answered on a 5-point Likert scale ranging from 1 (never) to 5 (very often). The cutoff score for this scale was 32, with a total score of 45. The criteria proposed in the literature were used to determine the cutoff score (35). Even though there is a cutoff, the scores obtained were examined along a continuum from casual gaming to gaming disorder. Cronbach's alpha of the scale for this study was strong i.e., 0.86, indicating good reliability.

#### 2.2.4. Rosenberg self-esteem scale

This 10-item unidimensional scale, developed by sociologist Dr. Morris Rosenberg in 1965 (36), was used to measure both positive and negative feelings about the self. It uses a scale of 0–30, where a score < 15 may indicate problematic low self-esteem. All items were answered using a 4-point Likert scale ranging from “strongly agree” to “strongly disagree.” In this study, Cronbach's alpha for the scale was 0.79, indicating good reliability.

#### 2.2.5. Brief sensation-seeking scale (BSSS)

The BSSS is an eight-item measure created by Hoyle et al. (37) to assess sensation-seeking among teenagers. This study used a culturally adapted version of the scale, which was validated in the English language in the Asian population (38). The response format follows a 5-level Likert-type scale. Scores ranged from 1 “strongly disagree” to 5 “strongly agree.” Higher scores indicate higher sensation-seeking behavior among participants. Cronbach's alpha value for this study was 0.70, showing adequate reliability.

### 2.3. Statistical analyses

IBM SPSS version 26 for Windows was used for statistical analysis of the data obtained, applying a significance level of a *p*-value of < 0.05. Descriptive statistics were carried out for data belonging to the socio-demographic sheet. In that, for continuous variables, the mean (M) and the standard deviation (SD) was computed, while the categorical variables were expressed as frequencies and percentages (%). The Kolmogorov–Smirnov test was used to determine whether the data were normally distributed. It was observed that data for both the independent and dependent variables were not normally distributed. Hence, non-parametric statistics were applied after that. The Spearman test was employed to find the correlation between all the variables, i.e., total scores of IGDS9-SF, SDQ, Rosenberg self-esteem scale, and BSSS-8, and the domain scores of SDQ. The effect size was based on the estimates given by Davis (1971), where *r* = 0.01–0.09 shows negligible correlation; *r* = 0.1–0.29 denotes low correlation; *r* = 0.3–0.49 shows moderate correlation; *r* = 0.5–0.69 shows substantial correlation; and *r* > 0.7 shows very strong correlation (39). Further, the Mann–Whitney *U*-test (MWU) was performed to see the difference of scores on all independent variables between female ‘with gaming disorder' and ‘without gaming disorder' as the gaming disorder variable data were non-normal. To ascertain the factors that predicted gaming disorder among female subjects, quantile regression was computed, an extension of linear regression used when the dependent variable shows a highly skewed distribution. This permitted us to model the relationships of the independent variables at different quantiles of the non-normally distributed dependent variable. The regression coefficients were estimated at five quantiles, 0.1, 0.25, 0.5, 0.75, and 0.9, based on the options available in the SPSS software.

## 3. Results

### 3.1. Socio-demographic profile of participants

A total of 707 adolescent girls participated in the study, with ages ranging from 12 to 19 years and the mean age and S.D. being 15 ± 1.5 years. These female participants were selected from school settings belonging to grades 8–12. Approximately 88% of participants had smartphones, 68% of the girls had a personal computer, 51% used tablets, and gaming consoles were owned by 22% of the subjects. Almost half of the participants were allowed to use these gadgets without the permission of their parents. Approximately 42% of participants had both their parents working, only 19% of them were single children, 8% of people came from single-parent households, whereas most of them (70%) belonged to nuclear families. The majority of the participants i.e., ~89% belonged to middle socio-economic status and the remaining 10% were from higher economic status. Only 1.5% of girls secured below-average scores on academic performance (< 40% in exams) while the rest had adequate academic performance. Approximately 96% of the girls also engaged in other extra-curricular activities such as hobbies or physical exercise and 5% of them have been reported to be using the substance at some point. A few participants also mentioned having physical and mental health conditions (4 and 3%), respectively, and three girls reported being diagnosed with gaming/online addiction.

Approximately 45% of participants in the sample reported playing games, most girls played games 1–3 times per week (34%) and nearly 6.5% engaged in gaming activities daily. Regarding hours spent gaming, 52% of female gamers played for < 2 h. Generally, those who played games played for < 2 h and very few proportions of participants, i.e., 2 and 0.3%, respectively, played games for 2–6 h or >6 h in a day. The adolescents reported physical discomfort, sleep disturbance, and delay in their food due to gadget use. Half of the participants had rules regarding gadget use being imposed by their parents, and they had a parental lock for what they browse on the Internet. Approximately 36% of girls experienced bullying in school, and many of them (83%) found academics stressful. The participants also found it difficult to utilize their time for study-related tasks and played games to overcome loneliness.

### 3.2. Trends of gaming among females: correlations and predictors

A total of 589 participants completed the measure of IGD, and the scores obtained showed low mean scores among the participants, i.e., an average of 12 out of 45. Total IGD scores were correlated with other psychological variables administered in the study: total Rosenberg self-esteem scores, total sensation-seeking (BSSS) scores, total scores on SDQ and domains of SDQ, namely “emotional symptoms,” “conduct,” “hyperactivity,” “peer problem” and “pro-social.” The result indicated that almost all the variables (total SDQ, total self-esteem, emotional symptoms, conduct, hyperactivity, and peer problem) were significantly correlated with IGD scores (see Table 2) at the 0.01 significance level, while sensation-seeking scores were significant at the 0.05 level and the pro-social domain was tending to be significant at the same level (*p* = 0.54). However, the correlation strength for these variables was negligible to low (*r* = 0.1–0.29). Scores on the IGD were positively correlated with the following variables: total SDQ, emotional symptoms, conduct, hyperactivity, peer problem, and sensation seeking but negatively correlated with variables pro-social behavior and self-esteem.

**Table 2:** Correlations between IGD scores and independent variables.

**Variable**	**Spearman's ρ_(n − 2)_*, p*-value**
Emotional symptoms	ρ_(584)_ = 0.172, *p* < 0.001^**^
Conduct problems	ρ_(581)_ = 0.292, *p* < 0.001^**^
Hyperactivity	ρ_(579)_ = 0.244, *p* < 0.001^**^
Peer-problems	ρ_(579)_ = 0.206, *p* < 0.001^**^
Prosocial behavior	ρ_(572)_= −0.080, *p* = 0.054
Total SDQ	ρ_(553)_ = 0.281, *p* < 0.001^**^
Total ROS score	ρ_(562)_ = −0.219, *p* < 0.001^**^
Total BSSS score	ρ_(573)_ = 0.104, *p* < 0.013^*^

SDQ, Strengths and Difficulties Questionnaire; ROS, Rosenberg Self-esteem Scale; BSSS, Brief Sensation Seeking Scale.

^*^Significant within p < 0.05.

^**^Significant within p < 0.01.

As the IGD scores did not follow a normal distribution (Kolmogorov–Smirnov test, *p* < 0.001), the relationship between the independent variables (total SDQ, total self-esteem, total sensation seeking-BSSS scores, emotional symptoms, conduct, hyperactivity, peer problem, and pro-social) with the total IGD score was analyzed using the quantile regression approach. Quantile regression allows modeling the relationships with different quantiles of the dependent variable that follows a skewed distribution. The regression coefficients were estimated at five quantiles: 0.1, 0.25, 0.5, 0.75, and 0.9.

**Figure 1:**
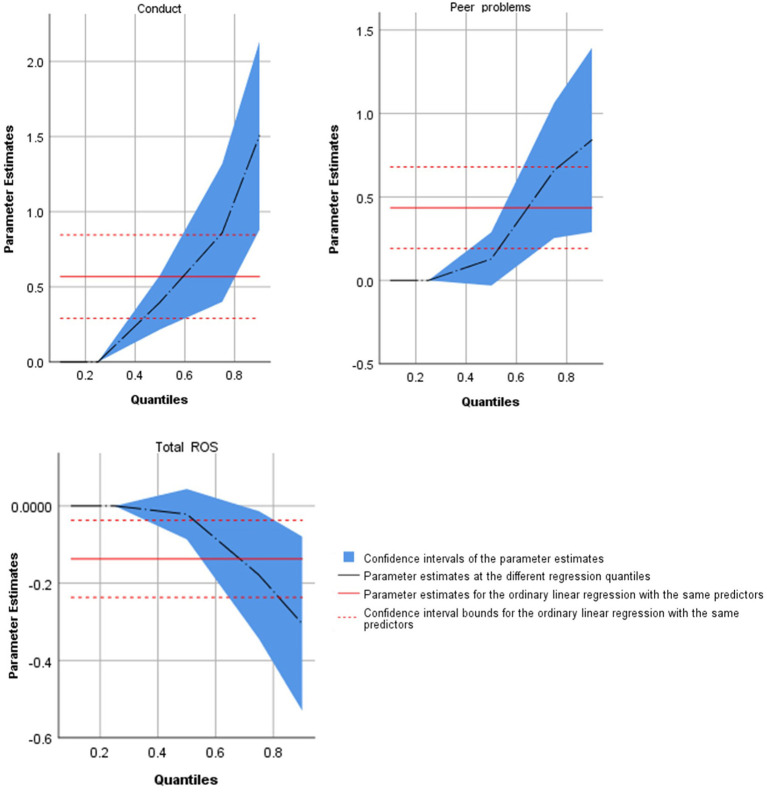
Parameter estimates of conduct, peer problems, total Rosenberg self-esteem scale score (total ROS) from quantile regression, and 95% confidence intervals.

At quantile 0.1 or the 10th percentile, hyperactivity and BSSS scores showed a significant (*p* < 0.05) but small effect (coefficient estimates in the order of 10^−17^) on the total IGD scores. At quantile 0.25 or the 25th percentile, none of the variables showed a significant effect on IGD scores. Conduct problems significantly predicted the 50th percentile of IGD scores [coefficient = 0.398, *t*_(520)_ = 4.31, *p* < 0.001], with stronger positive effects on 75th [coefficient = 0.859, *t*_(520)_ = 3.67, *p* < 0.001] and 90th [coefficient = 1.506, *t*_(520)_ = 4.72, *p* < 0.001] percentiles. Peer problems and Rosenberg self-esteem scale scores had significant positive and negative effects, respectively, on the IGD scores at quantiles 0.75 and 0.90 (*p* < 0.05). See Figure 1 for the coefficient estimates for the plots of parameter estimates with their 95% confidence intervals across quantiles.

**Figure 2:**
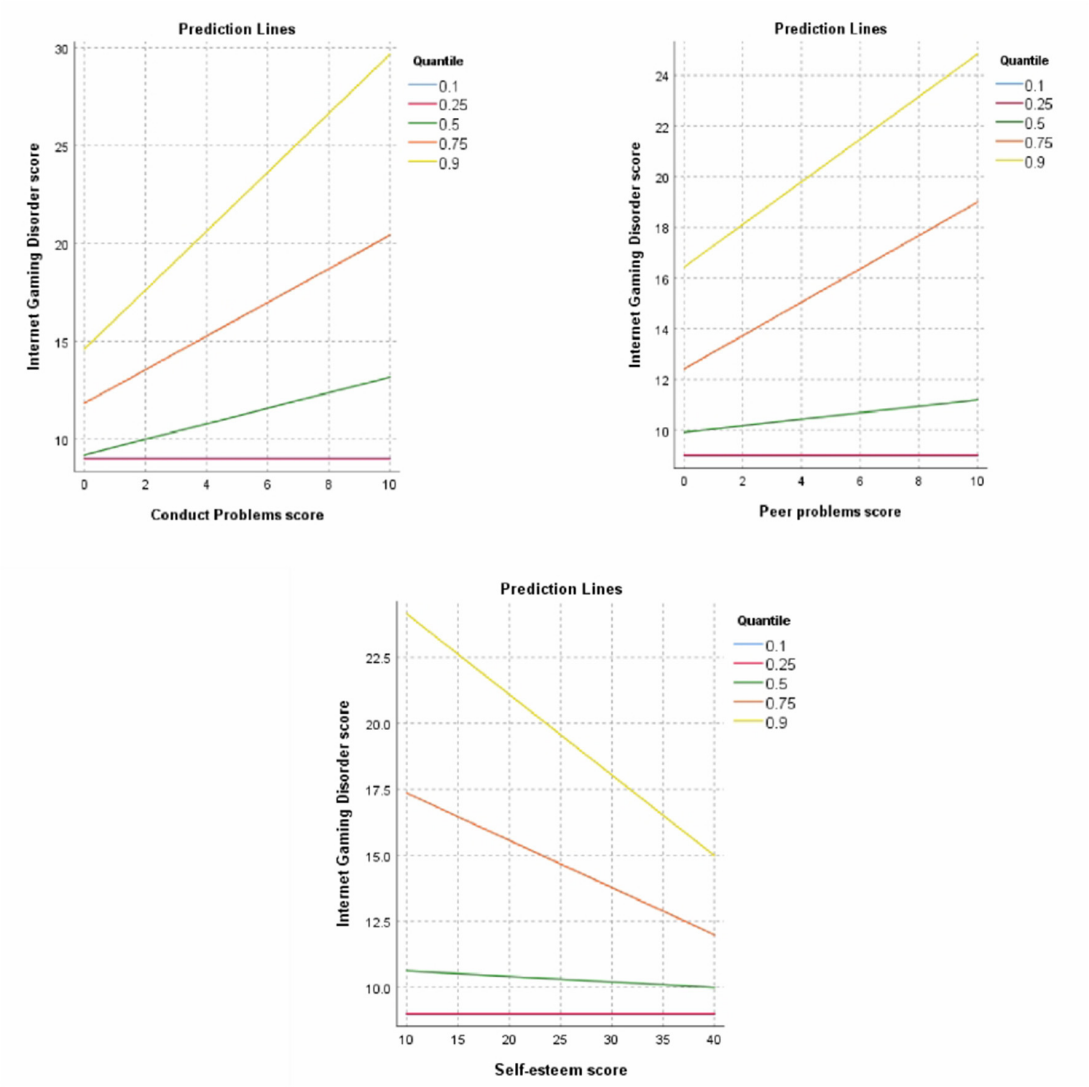
Prediction lines from quantile regression analysis show the relationships between the total Internet Gaming Disorder (IGD) scores and the variables that emerged significant in quantile regression, viz., conduct problems, peer problems, and total Rosenberg self-esteem (ROS) scores. The total IGD scores showed a positive relationship with conduct and peer problems, and an inverse relationship with self-esteem scores, which were stronger toward the higher quantiles.

In conclusion, conduct problems had a significant positive effect on the 50th percentile of IGD scores, with the effect becoming stronger at higher quantiles. Other variables with a significant effect on IGD scores included peer problems and Rosenberg self-esteem scores at quantiles 0.75 and 0.9. Thus, the positive relationship between conduct and peer problems on the IGD scores and the negative association with self-esteem scores became substantially stronger toward the higher quantiles, as shown in the graphs (Figure 2).

### 3.3. Comparison between gaming in participants “with gaming disorder” and “without gaming disorder”

Based on the cutoff score for the IGD scale (32 out of 45), only 0.8% subsample (five participants) among the sample was potentially having a gaming disorder. Using the cutoff score for IGD, the participants were divided into two groups: “with gaming disorder' and “without gaming disorder.” The Mann–Whitney *U*-test was applied to these two groups to compare them across all the independent variables. The comparison of both the groups showed significant differences across the following variables: total SDQ, total self-esteem scores, emotional symptoms, conduct, hyperactivity, and peer problem. Further a *post-hoc* analysis done by comparison of median scores of both groups on the significantly different variables (see Table 3) displayed that the “with gaming disorder” female group had significantly higher scores on emotional symptoms, conduct, hyperactivity, peer problem, and total SDQ scores, and on the other hand significantly lower scores on the Rosenberg self-esteem scale as compared to “without gaming disorder” female subjects.

**Table 3:** Comparison between groups of participants with and without gaming disorder.

**Variable**	**Without gaming disorder (*n* = 581)**	**With gaming disorder (*n* = 5)**	**Mann-Whitney test**
	**Median**	**Median**	***U***, ***z***, ***p*****-value**
	**Mean** ±**SD**	**Mean** ±**SD**	
Emotional symptoms	4.0	7.0	*U* = 485, *z* = −2.6, *p* = 0.010*
	3.9 ± 2.5	7.2 ± 2.4	
Conduct problems	2.0	5.0	*U* = 218.5, *z* = −3.3, *p* = 0.001*
	2.6 ± 1.6	5.6 ± 1.5	
Hyperactivity	4.0	9.0	*U* = 101.5, *z* = −3.2, *p* = 0.001*
	3.7 ± 2.1	8.5 ± 1.9	
Peer-problems	2.0	5.0	*U* = 433.5, *z* = −2.7, *p* = 0.006*
	2.5 ± 1.7	5.6 ± 2.5	
Prosocial behavior	8.0	8.0	*U* = 1,001.0, *z* = −1.2, *p* = 0.247
	7.7 ± 1.8	5.6 ± 3.9	
Total SDQ	20.0	31.5	*U* = 160.0, *z* = −3.0, *p* = 0.003*
	20.5 ± 5.6	31.8 ± 5.5	
Total ROS score	28.0	15.0	*U* = 116.0, *z* = −3.5, *p* < 0.001*
	28.3 ± 4.7	16.8 ± 4.7	
Total BSSS score	28.0	28.0	*U* = 1,325, *z* = −0.3, *p* = 0.786
	28.0 ± 4.9	26.6 ± 6.3	

SDQ, Strengths and Difficulties Questionnaire; ROS, Rosenberg Self-esteem Scale; BSSS, Brief Sensation Seeking Scale. * Significant within p < 0.01.

The socio-demographic profiling of the “with gaming disorder” group revealed that most of them belonged to nuclear families and secured adequate academic performance. However, three females from the group of five participants either belonged to single-parent households, secured below-average exam scores, or used substances. All participants reported physiological and biological consequences of physical discomfort and disrupted food and sleep routines due to excessive playing. It was common among them to argue with their parents regarding gadget use, and they found their parents use gadgets as well and spend more time on gadgets than interacting with them. The ‘gaming disorder' group had more friends online than in real, had experienced bullying in school at some point, found it difficult to concentrate on studies, and was stressed due to academics. They found no other activity as enjoyable as gaming; for all of them, gaming activities helped them overcome their loneliness.

## 4. Discussion

The research on gaming behavior and addiction has largely been biased toward the male population. There has been a significant dearth in the literature regarding generic gaming behavior and associated psychopathology related to problematic patterns in the female population. As noted in the literature, female gamers are taking up space in the gaming community (28), proactively participating in competitive games alongside male gamers and simultaneously improving their gaming skills. This study purports to bridge the research gap regarding gaming behavior among females, especially the adolescent age group (40). The study aimed to determine the association of problematic gaming with emotional and behavioral difficulties among female adolescents. The study's objectives were (i) to identify the trends and patterns of Internet gaming among female gamers and (ii) to assess the role of emotional and behavioral difficulties in female gamers as a predictive factor for problematic gaming.

The socio-demographic profile of the sample indicated that they predominantly belonged to middle socio-economic status, secured average academic performance, and regularly engaged in extra-curricular activities such as physical sports, exercise, or hobbies. It was noted that the main gadget possessed by the participants was a smartphone, followed by a personal computer/laptop. While 42% of the participants were using their parents' smartphones with their consent, 58% used them for personal reasons without their parents' consent. Another study done at the district level among adolescents in India quoted similar findings. They mentioned that 43% of adolescents used their parents' mobile phones to play games, and 57% of them played online games using their personal smartphones (41). Gaming consoles were owned by a quarter of the participants (22%). The ownership of gaming consoles recorded in this sample is significantly lower than the statistics provided by a US survey, which claimed that 47% of adolescent girls and 56% of teenage boys possessed portable consoles (42).

The result further showed that 47% of girls never played games and 40% of participants reported that they played games although the gaming frequency is relatively low, with most of them stating that they play games 1–3 times a week for < 2 h. Nearly 7% of people from the total sample of girls played games daily. This suggests that gaming is not a common habit among female players in this group. Similar findings were reported by research among the Swedish adolescent population for both male and female gamers. The conclusions of that study showed among female adolescents, only 37% played games and most of them who played games engaged in gaming for < 2 h a day (43).

In this study, 0.8% of the participants met the criteria for gaming disorder according to the IGD scale. This finding is consistent with previous research focusing on female gamers, which found that only 1% of the 514 female gamers had potential IGD (24). Another meta-analysis research on the prevalence of female gaming disorder shows the rate to be 1.3%, representing the low rate of gaming disorder among female adolescents.

However, the same study quoted prevalence among male adolescents as 6.5%. This indicates that male gamers have a higher prevalence of gaming disorders than female gamers (19). As discussed above, female gamers stop gaming earlier than their male counterparts due to negative online experiences and emotional difficulties. Hence, as a consequence, their gaming pattern might not reach the level of dysfunction. As previously stated, women are more likely to be casual gamers who play less competitive games. The latter requires more time investment and rigorous training, possibly leading to dysfunctional gaming behavior.

Western studies and statistical data quote that the proportion of female gamers has increased by many times over the years, with 61% of women in the UK (44) and 48% in the US playing games (45) in the year 2021. Moreover, it has been found that female participation in gaming increases with age as, according to an ESA report, women aged 45+ spent nearly 11 h a week playing (45). This indicates that future studies in female gaming will require a diverse sample representation to determine whether age is a useful variable in predicting problematic gaming among female gamers.

In addition, 4 and 3% of participants, respectively, reported having physical and mental health symptoms. Along similar lines, the prevalence study conducted among Indian adolescents shows that 7% of adolescents have mental health problems (46). On the contrary, a few Western research data showed that 28–33% of adolescents (47, 48) have physical health conditions that are much higher than the prevalence rate in this study. Another study investigating the physical health of adolescents in India found that 63% of boys and 42% of girls were undernourished (49). The low statistical data reported in this study could be attributed to the lack of a standardized screener for evaluating physical and mental health disorders.

In this study, gaming disorder and the SDQ-measured symptoms of emotional and behavioral difficulties, as well as sensation seeking and self-esteem, all significantly correlate with one another. Much research has investigated the relationship between gaming/internet addiction and SDQ scores. All these studies showed a favorable relationship between gaming and the psychopathological dimensions of emotional and behavioral issues in SDQ (50–52). This finding was confirmed in the current study.

Regarding the psychological variable of self-esteem, such as the results obtained in this research, a prior study found a significant negative correlation between gaming disorder and self-esteem (53). However, a different study established a positive relationship between these two characteristics (54). Meanwhile, other available literature confirms the positive relationship between sensation-seeking and gaming addiction which is mediated by other affective factors such as self-control, neuroticism, anxiety, and impulsivity (55, 56).

The literature review demonstrates that the factors examined in this study are relevant to gaming disorder and are also valid for the male population. Although there was a correlation between the gaming disorder score and all the research variables, it was either negligible or weak. Therefore, the results of this finding should be interpreted with caution.

It was noted that participants falling under the category “with gaming disorder” had experiences of bullying in school, had fewer friends in the real world, and perceived academics as stressful. Few recent studies established a positive association between gaming and social bullying on online platforms (57, 58). Concerning perceived stress and gaming, previous literature supports the bi-directional relationship between these two variables for the adolescent population (59) and loneliness as a gravitating factor toward disordered gaming among them (60). Furthermore, researchers have also shown several physiological and psychological consequences, such as body pain, strain in the eyes, sleep disturbance, irregular eating habits, inattention, cognitive difficulties (61, 62), and academic decline (63) as a result of excessive gaming; these findings also resonated with the results of the current study.

In the current research, a trend-level relationship was primarily observed between problematic gaming, behavioral difficulties of conduct, peer problems, and emotions related to the self-esteem issue. The finding indicates an association between gaming disorder and emotional and behavioral difficulties, further mediated by low self-esteem. In previous studies, respective to at-risk gamers in a clinical setting, it was found that addicted adolescent gamers frequently experienced conduct problems, hyperactivity/inattention issues, and emotional, behavioral, and peer-related issues. The same psychological variables were also predominant among male gamers with a gaming problem. Furthermore, the study finding revealed that addicted female gamers had considerably more behavioral, emotional, and peer problems than addicted male gamers and casual and regular female gamers (32). The current research discovered similar findings of psychopathological features among at-risk female gamers. This may indicate that gaming disorder among females can both be a consequence as well as a cause of severe psychological difficulties manifested in the form of behavioral and emotional symptoms mediated by several social factors such as cultural role expectation, media portrayal of gaming as highly rewarding, expanding gaming business through monetary gains, peer-norms, and general social isolation of the current generation.

## 5. Limitations and implications

For this study, convenience-based sampling was used as the research was carried out during the pandemic when schools were closed for in-person classes. The researcher met with significant challenges at every step of data collection and ensuring data completion from the participants.

Self-report scales were administered to the participants to gather information regarding gaming patterns and psychological variables. However, augmenting the study with a clinician-based rating scale would have provided a subjective and objective understanding of the problem we wanted to study.

The research was carried out on a community sample; therefore, a stringent exclusion criterion was not kept, but screening for intellectual disability and having standardized measures for psychological disorders and physical disease among participants would have added to the rigor of the research.

The present trend shown in the research on psychopathological characteristics among at-risk female gamers requires a larger sample size for validation as in this study few participants (*n* = 5) met the criteria for gaming disorder. Since the study has specifically targeted the female adolescent population, the generalizability of the finding related to the association of emotional and behavioral difficulties with gaming disorder is questionable. However, a similar trend has also been observed among male adolescents. This study focused on the generic understanding of female adolescent gaming patterns and their related psychopathological characteristics. Hence, specific attributes related to motivations and barriers to playing games, information about gaming genres, and experience as female gamers in the male-dominated gaming world have not been explored. There is a need to measure such attributes in further studies using quantitative and qualitative methods in the Indian context to understand trends and patterns of gaming among female gamers. A more varied representative sample of female gamers with a wider age group and SES would have been useful in providing a detailed understanding of gaming among this population. Thus, future studies require a large female sample cross-sectional with varied socio-demographic representation and probability-based sampling strategy.

Furthermore, the knowledge gained from this study regarding the psychopathological problems of at-risk female gamers can help us develop a theoretical module that can contribute to early screening and evolving preventive strategies that can mediate female gamers from exhibiting potentially addictive tendencies.

## 6. Conclusion

The study conveys that behavioral symptoms of conduct, peer problems, and emotional symptoms of self-esteem predicted trend-level problematic gaming among female adolescents. Therefore, with higher conduct, peer-related problems, and lower self-esteem, there are greater tendencies for probable gaming disorder.

## Data availability statement

The raw data supporting the conclusions of this article will be made available by the authors, without undue reservation.

## Ethics statement

This study was reviewed and approved by the Ethics Committee (Behavioral Sciences Division) NIMHANS-NIMH/DO/BEH. Sc. Div./2021-22. Written informed consent to participate in this study was provided by the participants' legal guardian/next of kin.

## Author contributions

PT, MS, JK, and NA contributed to the conceptualization, methodology, and validation. PT, VM, and PM performed formal analysis. PT performed the investigation and did writing—original draft preparation. PT and VM conducted data curation and did the visualization. MS performed writing—review and editing. All authors contributed to the article and approved the submitted version.
